# Error in Figure Captions

**DOI:** 10.1001/jamanetworkopen.2021.27643

**Published:** 2021-08-25

In the Original Investigation titled “Comparison of Lemborexant With Placebo and Zolpidem Tartrate Extended Release for the Treatment of Older Adults With Insomnia Disorder: A Phase 3 Randomized Clinical Trial,”^1^ published December 27, 2019, there was a numeric reporting error in the caption to Figure 2 and Figure 3. In both captions, the phrase “A total of 208 participants received placebo, 263 received 6.5 mg of zolpidem tartrate extended release” should have been “A total of 208 participants received placebo, 263 received 6.25 mg of zolpidem tartrate extended release.” This article has been corrected.^1^ This article was previously corrected to fix an error in a trial registration number in the Abstract.^2^
